# 3,5-Diiodo-L-Thyronine Administration To Hypothyroid Rats Rapidly Enhances Fatty Acid Oxidation Rate and Bioenergetic Parameters in Liver Cells

**DOI:** 10.1371/journal.pone.0052328

**Published:** 2013-01-04

**Authors:** Alessandro Cavallo, Paola Priore, Gabriele Vincenzo Gnoni, Sergio Papa, Franco Zanotti, Antonio Gnoni

**Affiliations:** 1 Laboratory of Biochemistry, Department of Biological and Environmental Sciences and Technologies, University of Salento, Lecce, Italy; 2 Institute of Biomembranes and Bioenergetics, National Research Council (CNR), Bari, Italy; 3 Department of Basic Medical Science, University of Bari “Aldo Moro”, Bari, Italy; Pennington Biomedical Research Center, United States of America

## Abstract

Growing evidence shows that, among triiodothyronine derivatives, 3,5 diiodo-L-thyronine (T_2_) plays an important role in energy metabolism and fat storage. In the present study, short-term effects of T_2_ administration to hypothyroid rats on fatty acid oxidation rate and bioenergetic parameters were investigated. Within 1 h following T_2_ injection, state 3 and state 4 respiration rates, which were reduced in hypothyroid mitochondria, were noticeably increased particularly in succinate- with respect to glutamate/malate-energized mitochondria. Maximal respiratory activity, observed when glutamate/malate/succinate were simultaneously present in the respiratory medium, was significantly stimulated by T_2_ treatment. A T_2_-induced increase in respiratory rates was also observed when palmitoyl-CoA or L-palmitoylcarnitine were used as substrates. No significant change in respiratory control index and ADP/O ratio was observed. The activities of the mitochondrial respiratory chain complexes, especially Complex II, were increased in T_2_-treated rats. In the latter, Complex V activities, assayed in both ATP synthesis and hydrolysis direction, were enhanced. The rate of fatty acid oxidation, followed by conversion of [^14^C]palmitate to CO_2_ and ketone bodies, was higher in hepatocytes isolated from T_2_-treated rats. This increase occurs in parallel with the raise in the activity of carnitine palmitoyltransferase-I, the rate limiting enzyme of fatty acid β-oxidation, assayed *in situ* in digitonin-permeabilized hepatocytes. Overall, these results indicate that T_2_ rapidly increases the ability of mitochondria to import and oxidize fatty acids. An emerging idea in the literature is the ability of T_2_ to reduce adiposity and dyslipidemia and to prevent the development in liver steatosis. The results of the present study, showing a rapid T_2_-induced increase in the ability of mitochondria to import and oxidize fatty acids, may contribute to understand the biochemical mechanisms of T_2_-metabolic effects.

## Introduction

The thyroid hormones (TH) 3,3′,5,5′-tetraiodo-L-thyronine (T_4_) and 3,3′,5-triiodo-L-thyronine (T_3_) exert significant actions on energy metabolism; mitochondria, by virtue of their biochemical functions, are key cellular sites for TH metabolic effects [1]–[3]. Alterations of the thyroid state of animals have noticeable effects on the synthesis [2], the turnover and functional capacity of mitochondrial components [4]. Liver mitochondria from hypothyroid rats show a decreased activity of membrane-associated electron transport enzymes and anion carriers [5]–[9], a failure that has been ascribed to a lower expression of their corresponding proteins. Genomic and non genomic effects are associated with TH actions [1]–[3]. In particular, TH may modify the expression of some respiratory genes [10], [11] and other genes involved in ATP producing and consuming functions [11], as well as in fatty acid oxidation [12]. However, the molecules mediating non genomic effects of TH remain to be fully identified [13]. It has been demonstrated that iodothyronines other than T_3_ and T_4_ can play a physiological role. Several evidences indicated that 3,5-diiodo-L-thyronine (T_2_), previously considered as simple degradation product of T_3_, appears to influence energy metabolism [1], [14], [15]. T_2_ is able to stimulate the mitochondrial oxidative capacity and respiration rate in mammals [1], [11], [14], [15]. Similar results have been obtained on isolated mitochondria from fish [16], indicating that such effects are not restricted to mammalian species. Animal cells generate most of their ATP by oxidative phosphorylation (OXPHOS), a process located in the inner mitochondrial membrane. We have recently shown that the acute T_2_ administration to hypothyroid rats increases F_o_F_1_-ATP synthase activity in liver mitochondria [15]. T_2_ effects on different metabolic pathways have been shown to be more rapid than those of T_3_ and often independent of protein synthesis [15]–[19].

T_2_ ability to affect whole animal metabolic rate is of growing interest. It has been shown that T_2_ is able to rapidly increase the resting metabolic rate of hypothyroid rats [20], and to powerfully reduce adiposity in rats fed high-fat diet by increasing lipid degradation [21]. A direct action of T_2_ in commutating hepatic steatosis, a condition commonly associated with diet-induced obesity, has been demonstrated [22]. The addition of T_2_ to fat-overloaded rat hepatocytes resulted in a reduction of lipid content and lipid droplets (LD) diameter together with an improvement of the oxidative stress conditions induced by excess lipids in the cells [23]. However, T_2_ modulation of LD metabolism still remains unsolved. In parallel, *in vivo* studies demonstrated that T_2_ chronic administration may stimulate hepatic fat reduction in rats fed a high-fat diet through the increased oxidation of fats [24], [25].

Carnitine palmitoyltransferase–I (CPT-I), the mitochondrial gateway for fatty acid entry into the matrix, is the main controller of the hepatic mitochondrial β-oxidation flux [26]. CPT-I, located in the outer mitochondrial membrane, transfers the fatty-acyl moiety from acyl-CoA to carnitine. The acylcarnitine formed is transported across the inner mitochondrial membrane by carnitine acylcarnitine translocase and reesterified to acylCoA by CPT-II. In the liver, CPT-I exerts approximately 80% of control of fatty acid β-oxidation rate under physiological conditions [26].

Several evidences indicated that T_2_ administration to hypothyroid rats increases mitochondrial fatty acid oxidation rate [27], [28] and thermogenesis in rat skeletal muscle [27]. However, information concerning the ability of T_2_ to modulate hepatic fatty acid degradation are lacking [21] in spite of the fact that liver represents a major contributor to energy expenditure [29].

Considering that T_2_ is able to reduce hepatic lipid accumulation in several high-fat experimental models [21]–[25] and in the light of our recent findings on the stimulating effect of this hormone on F_o_F_1_-ATP synthase activity in rat liver [15], we decided to investigate the biochemical mechanisms underlying the capacity of T_2_ to affect lipid degradation. To this end we studied in rat-liver cells the effect of T_2_ on the complete pathway of fatty acid oxidation from CPT-I activity and fatty acid oxidation rate to the downstream pathways such as mitochondrial OXPHOS activities.

The present study provides direct evidence that CPT-I activity and the total rate of fatty acid oxidation, both reduced in liver of hypothyroid as compared to euthyroid rats, were rapidly (within 1 h) increased by T_2_ administration, as shown by the raise in CO_2_ and ketone bodies production. This increase was associated with a parallel enhancement of OXPHOS activities.

## Materials and Methods

### Ethics Statement

All rats received care in compliance with the Principles of Laboratory Animal Care formulated by the National Society for Medical Research and the Guide for the Care and Use of Laboratory Animals prepared by the Institute of Laboratory Animal Resources, published by the National Institutes of Health (NIH Publication No. 86-23, revised 1985), as well as in in accordance with Italian laws on animal experimentation (art. 4 and 5 of D.L. 116/92).

### Animal treatment

Male Wistar rats (200–250 g) were housed one for cage in a temperature- (22±1°C) and light- (light on 08:00h–20:00h) controlled room. A commercial mash and water were available *ad libitum*. T_2_ and iopanoic acid (IOP) were dissolved in 0.05M NaOH and diluted with 0.09% NaCl. At the start of the study the rats were subjected to a week of acclimatization. Three groups of rats were used throughout: i) euthyroid control rats were treated with only vehicle for 3 weeks; ii) rats were made hypothyroid by 6-n-propyl-2-thiouracil (PTU) administration in the drinking water (0.1% w/v) for three weeks together with a weekly i.p. injection of IOP (6 mg/100 g bw); iii) at the end of this treatment, hypo+T_2_ rats received a single i.p. injection of T_2_ (150 µg/100 g bw). This dose of T_2_ has been shown to produce a clear-cut, rapid effect on energy expenditure [15], [30]. 1 h after T_2_ injection, rats were anesthetized and killed by decapitation.

### Mitochondria isolation and oxygraphic measurements

Rat livers were rapidly processed and mitochondria isolated by differential centrifugation essentially as described [31]. Briefly, homogenization medium contained 250 mM sucrose, 10 mM Tris (pH 7.4), 1 mM EDTA, and 0.1% defatted bovine serum albumin (BSA; fraction V, fatty acid-free from Sigma), which was omitted in the final washing medium. The homogenate was centrifuged at 1,100× g for 8 min and mitochondria were precipitated from the supernatant at 7,700× g for 10 min. Mitochondrial pellet, suspended in the homogenization medium, was centrifuged twice at 12,100× g for 10 min and finally resuspended in the washing medium. The obtained mitochondrial suspension, devoided of peroxisomes as judged from measurements of recovered catalase activity (results not shown), was immediately used for the subsequent experiments. To assess the functional integrity of isolated mitochondria, their respiration rate was measured by a Clark-type oxygen electrode. Mitochondrial respiration (0.3 mg mitochondrial protein/ml) was measured in a medium containing 220 mM sucrose, 20 mM KCl, 2.5 mM KH_2_PO_4_, 1 mM EDTA, 20 mM HEPES, 5 mM MgCl_2_, 0.1% BSA. Mitochondrial respiration in state 3, (indicative of the coupled respiration in which phosphorylation of ADP is at the maximal rate) was initiated by the addition of 0.3 mM MgADP and followed until the total consumption of ADP, when state 4 (resting state of respiration, measured in the presence of respiratory substrate alone and without ATP formation) was measured. Then, 4 mM carbonyl cyanide 4-(trifluoromethoxy) phenylhydrazone (FCCP) was added to measure uncoupled respiration. State 3, state 4, uncoupled respiration, respiratory control index (RCI, state 3/state 4 ratio) and ADP/O ratio (ADP consumed/oxygen atoms consumed during the whole state 3 period) were evaluated as in [32]. Protein was determined by the Bradford method using BSA as the reference standard.

The ability of T_2_ to affect FADH_2_-linked respiratory pathways was followed by mitochondrial oxidation of succinate 5 mM (Complex II), in the presence of rotenone, while glutamate 5 mM/malate 2.5 mM were used as respiratory substrates to investigate T_2_ effect on NADH-linked respiratory pathways (Complex I). Maximal mitochondrial respiration depends on convergent electron flow through Complex I+Complex II to the ubiquinone-junction of the electron transport system. In order to investigate maximal respiratory capacity of mitochondria, a mixture of glutamate 5 mM/malate 2.5 mM/succinate 5 mM (without rotenone) as respiratory substrates was used in some experiments. This mixture is closer to *in vivo* condition than when using only one type of substrate. In this context, all electron transports within respiratory chain are possible (for review see [33]). T_2_ ability to affect mitochondrial capacity to oxidize fatty acids was assessed by using, as respiratory substrate, palmitoyl-CoA or L-palmitoylcarnitine. For palmitoyl-CoA oxidation, incubation medium (3.0 ml) was supplemented with 2 mM carnitine, 2.5 mM malate and 0.2 mM ADP and mitochondria (1 mg protein/ml) were energised with 40 µM palmitoyl-CoA, while to detect mitochondrial L-palmitoylcarnitine oxidation, incubation medium was supplemented with 2.5 mM malonate, 2 mM carnitine, 0.5 mM ADP, and the reaction was started by the addition of 40 µM L-palmitoylcarnitine as reported in [27].

### Activity assays of the oxidative phosphorylation complexes

Enzymatic activities of respiratory chain complexes (I, II–III, IV) were assayed essentially as in [34] with slight modifications. Briefly, *NADH: CoenzymeQ oxidoreductase* (Complex I, EC 1.6.5.3) activity was assayed spectrophotometrically by measuring the NADH absorbance decrease at 340 nm. Standard reaction medium (1.0 ml) was supplemented with 60 µM decylubiquinone, 0.1 µg antimycin A, 1 mM KCN and 0.5 mg proteins of broken mitochondria, with three cycles of freezing and thawing. The reaction was initiated by 100 µM NADH, and 2 min after 2 µM rotenone was added. Enzyme activity was determined as the difference of absorbance in the absence and in the presence of rotenone.

#### Succinate

*Coenzyme Q oxidoreductase* (Complex II, EC 1.3.5.1) activity was determined spectrophotometrically by measuring at 600 nm the reduction of 2,6-dichloroindophenol (DCIP) in a 1.0 ml final volume of reaction mixture containing 80 mM KH_2_PO_4_ buffer (pH 7.8), 1 g/l BSA, 2 mM EDTA, 0.2 mM ATP, 10 mM succinate, 3 µM rotenone, 0.1 µM antimycin A, 1 mM KCN, 50 µM decylubiquinone and 80 µM DCIP. 0.5 mg proteins of broken mitochondria were first preincubated in a buffer without KCN and succinate, which were added after 10 min and the absorbance was measured every minute for 5 min.

#### Succinate

*cytochrome c reductase* (Complex II–III, EC 1.10.2.2) was evaluated spectrophotometrically following the reduction of cytochrome C by the increase in absorbance at 550 nm. Reaction started by the addition of 5 mM succinate to 2.0 ml of the reaction medium containing 2 µM rotenone, 1 mM KCN, 60 µM cytochrome c, and 0.3 mg proteins of broken mitochondria.

*Cytochrome c oxidase* (Complex IV, EC 1.9.3.1) activity was determined polarographically in 1.0 ml reaction mixture containing 2 µM rotenone, 0.1 µg antimycin A, 10 µM cytochrome c, and 0.5 mg proteins of broken mitochondria. The reaction started by adding 5 mM ascorbate plus 0.25 mM TMPD (N,N,N′,N′- tetramethyl-p-phenylenediamide).

*F_o_F_1_-ATP synthase* (Complex V, EC 3.6.3.14) activity was determined on freshly isolated mitochondria, while olygomycin-sensitive ATP hydrolase activity was determined with an ATP-regenerating system in inside-out submitochondrial particles prepared in the presence of EDTA (ESMP) by exposure of mitochondria to ultrasonic energy [35].

Liver ATP content was assayed luminometrically using ATP Bioluminescent Assay Kit (Sigma St.Louis, MO, USA) according to the provided protocol. ATP µmoles were calculated according to a standard curve and related to g of liver.

### Isolation and incubation of hepatocytes

Hepatocytes were isolated by the collagenase perfusion method [35]. In order to minimize glycogenolysis, 20 mM glucose was added to the perfusion buffer and to all buffers subsequently employed in the experiments. Hepatocyte suspensions were incubated in Krebs-Henseleit bicarbonate buffer (pH 7.4) supplemented with 10 mM glucose. Incubations (4–6 mg of cellular protein/ml) were carried out in 25 ml Erlenmeyer flasks in a metabolic shaker (85 oscillations per min) at 37°C in a total volume of 2.0 ml under an atmosphere of O_2_/CO_2_ (19: 1 v/v).

### Rate of palmitate oxidation in isolated hepatocytes

Rate of labeled palmitate oxidation was determined in intact hepatocytes as formation of total oxidation products, i.e. CO_2_ and acid-soluble products (ASP), mostly constituted by ketone bodies [36]. Briefly, [1-^14^C] palmitate (0.5 mM, 0.1 Ci/mol), bound to BSA in a 5∶1 molar ratio, was added to hepatocyte suspensions at 37°C. After 20 min, reactions were stopped by the addition of 0.3 ml of 2 M perchloric acid. At the same time, 0.15 ml of benzethonium hydroxide (1 M in methanol) was injected in a center well containing filter paper. Samples were allowed to equilibrate for an additional hour at 4°C, and the center well content (with the ^14^CO_2_ fixed as bicarbonate) was transferred into vials for radioactivity counting. The cell precipitate was spun down, and supernatants were washed three times with light petroleum ether. ASP were subsequently extracted from the samples [36]. Total oxidation products were determined as the sum of radioactivity of CO_2_ plus ASP.

### Assay of CPT-I activity

CPT-I activity was determined in: whole liver homogenate, freshly isolated mitochondria, and digitonin-permeabilized hepatocytes, as the malonyl-CoA sensitive incorporation of radiolabeled L-carnitine into acylcarnitine using palmitoyl-CoA as substrate [36].

The CPT-I assay mixture for total homogenate (1 mg protein/ml) and isolated organelles (0.2 mg mitochondrial protein/ml) consisted of 25 mM Tris–HCl, pH 7.4, 150 mM sucrose, 60 mM KCl, 1 mM dithioerythritol, 2 mg/ml defatted and dialysed BSA, 0.4 mM L-[methyl-^14^C]carnitine (1 Ci/mol) and 50 µM palmitoyl-CoA. Reactions, proceeding linearly up to 4 min (data not shown), were carried out at 30°C for 2 min and stopped with 1 ml of 1.2M HCl. CPT activity that was insensitive to 100 µM malonyl-CoA was always subtracted from the CPT activity. This malonyl-CoA-insensitive CPT activity routinely accounted for 5–10% of the total CPT activity experimentally determined.

It has been demonstrated that the activity of liver CPT-I is affected by extramitochondrial cell components that are lost on isolation of mitochondria [37]–[39]. Permeabilizing cells with digitonin allows to assay rapidly intracellular enzyme activities under more or less physiological conditions and avoiding any possible post-homogenizing modifications, which can occur in subcellular fractionation [36]–[38]. In such a procedure cell permeabilization and assay of enzyme activity are simultaneously performed *in situ*. Permeabilization of hepatocytes was obtained with 4 µg/ml digitonin (a concentration chosen after titration with different digitonin concentrations) as reported [38]. The CPT-I activity was determined as specified above. The CPT-I assay mixture consisted of 12.5 mM Tris–HCl, pH 7.4, 70 mM sucrose, 30 mM KCl, 1.56 mM dithioerythritol, 0.5 mM L-[methyl-^14^C]carnitine (2 Ci/mol).

Reactions, proceeding linearly up to 1.5 min (data not shown), were carried out at 37°C for 40 s and stopped with 0.4 ml of 1M HCl. The reaction product, [^14^C]palmitoylcarnitine, was extracted with n-butanol [36].

### Determination of mtDNA copy number

Total DNA was obtained from 0.3 g of liver by phenol/chloroform extraction. Real-time quantitative PCR (SmartCycler System, Cepheid) using SYBR Green technology (FluoCycle, Euroclone) was performed to quantify mitochondrial DNA (mtDNA) content. The primers used in real time PCR were D-loopF1 ggTTCTTACTTCAgggCCATC and D-loopR1 TgACCTTCATgCCTTgACgg for the D-loop region of mtDNA (Gen-Bank™ accession number AC_000022.2); GAPDHFor 5′-ATCATACTTggCCTTgTAAg-3′ GAPDHRev 5′-AgggCAAAACCAAAgATgACTG-3′ for the nuclear gene *Gadph* (GenBank/NCBI Data Bank Accession no. NC_005103) [40]. A standard curve for each target sequence (mitochondrial and nuclear) was created using ten-fold serial dilutions of starting template DNA. The quantity (pg) of mtDNA and nuclear DNA (nDNA) was calculated from the corresponding standard curve. The results of the mtDNA level was expressed as mtDNA quantity to nuclear DNA quantity ratio (mtDNA/nDNA).

### Statistical analysis

Data are expressed as means ± standard deviation (SD). The unpaired t test was used to assess the significance of difference between means. P<0.05 was taken as the lowest level of significance.

## Results

In order to minimize potential interference by thyroid hormones, we chose to perform experiments by i.p. administration of T_2_ to hypothyroid rats. The experimental administration (PTU+IOP) we used to obtain hypothyroid state, the same as in Goglia's laboratory [19], [27], allows the observed effects to be directly attributed to the iodothyronine administered (T_2_).

### T_2_ rapidly enhances mitochondrial respiratory parameters

Table 1 shows that, using glutamate/malate as respiratory substrates, state 3 and state 4 respiration rates were reduced in hypothyroid mitochondria as compared to euthyroid and were enhanced in mitochondria from T_2_-treated rats.

**Table 1:** Oxygen uptake in liver mitochondria isolated from euthyroid, hypothyroid and hypothyroid plus T_2_-treated rats.

Rats	State 3	State 4	FCCP-stimulated	RCI	ADP/O
**Glutamate+Malate**					
Euthyroid	80.5±15^a^	11.3±3^a^	99.2±23^a^	7.1±2	2.5±0.1
Hypothyroid	58.0±9^b^	8.4±2^b^	74.2±18^b^	6.9±1	2.6±0.1
Hypothyroid+T_2_	70.2±15^a^	10.1±3^a^	91.2±15^a^	6.9±2	2.7±0.1
**Succinate**					
Euthyroid	156.7±10^a^	22.6±9^a^	207.7±21^a^	6.9±1	1.9±0.3
Hypothyroid	88.3±8^b^	13.3±2^b^	129.7±15^b^	6.6±1	2.0±0.2
Hypothyroid+T_2_	130.5±11^c^	21.4±2^c^	169.2±19^c^	6.1±1	1.9±0.1
**Glutamate+Malate+Succinate**					
Euthyroid	198.7±12^a^	26.9±6^a^	247.7±18^a^	7.4±2	2.3±0.2
Hypothyroid	117.3±10^b^	15.7±4^b^	169.3±12^b^	7.4±2	2.6±0.3
Hypothyroid+T_2_	158.1±12^c^	24.7±6^c^	205.6±21^c^	6.4±2	2.4±0.2

Glutamate(5 mM)+malate (2.5 mM), succinate (5 mM) or a mixture of glutamate+malate+succinate (5, 2.5, 5 mM) were used as respiratory substrates. Oxygen consumption was measured by a Clark oxygen electrode. Respiratory activity of state 3, state 4 and FCCP-stimulated was expressed as natoms oxygen/min/mg of mitochondrial proteins. Respiratory control index (RCI) represents the ratio between state 3 and state 4 respiration rate. Data are means ± SD of 6 experiments performed with duplicate samples. Values sharing a different letter differ significantly. P<0.05.

A similar, but more pronounced trend was observed in succinate-supported respiration. Consistent with previous data [6], [15], [32], [41], respiration rate from succinate was noticeably reduced in hypothyroid mitochondria. T_2_ administration showed vs the latter an increase in state 3 (48%) and state 4 (38%) oxygen consumption, which was higher than that observed in glutamate/malate-supported respiration. Similarly, maximal respiratory capacity of mitochondria, obtained in presence of glutamate/malate/succinate as respiratory substrates, was reduced by approx 40% in hypothyroid mitochondria, while was significantly raised in mitochondria from T_2_-treated rats. The addition of an uncoupler (FCCP) to mitochondria allows the evaluation of the electron respiratory chain function without the control exerted by F_o_F_1_-ATP synthase. The FCCP-stimulated uncoupled respiration was also promoted by T_2_.

RCI, which represents the ratio between state 3 and state 4 respiration, was similar (and higher than 6) in mitochondria isolated from euthyroid and treated rats, thus indicating a good quality of mitochondrial preparations. An efficient OXPHOS activity, as indicated by the ADP/O ratio values, was observed both with NAD-linked substrates (glutamate+malate) and FAD-linked (succinate) substrate.

The ability of T_2_ to affect fatty acid oxidation rate was assessed by using as respiratory substrate palmitoyl-CoA (+carnitine) or L-palmitoylcarnitine. Even if to a lesser extent with respect to succinate, T_2_ was able to increase fatty acid-supported mitochondrial respiratory activities, which were reduced in hypothyroid as compared with euthyroid mitochondria (Table 2).

**Table 2:** Effect of 3,5-diiodothyronine on rat-liver mitochondrial respiratory rates from lipid substrates.

Rats	State 3	State 4	FCCP-stimulated	RCI	ADP/O
**Palmitoyl-CoA+Carnitine**					
Euthyroid	28.1±4	6.5±2	43.0±8	4.3±0.7	2.6±0.2
Hypothyroid	21.6±5	5.0±1	30.8±6	4.3±0.8	2.4±0.4
Hypothyroid+T_2_	26.3±4	6.3±2	45.8±6	4.2±0.7	2.4±0.3
**L-Palmitoylcarnitine**					
Euthyroid	39.6±6	8.7±1	64.5±14	4.6±1.1	2.3±0.3
Hypothyroid	27.6±4	6.0±1	56.1±11	4.6±1.1	2.4±0.3
Hypothyroid+T_2_	29.3±4	6.8±2	62.0±12	4.3±1.3	2.3±0.2

Palmitoyl-CoA+carnitine or L-palmitoylcarnitine were used as respiratory substrates. Mitochondrial respiratory activity in state 3, state 4 and with FCCP is expressed as natoms oxygen/min/mg mitochondrial proteins. Respiratory control index (RCI) represents the ratio between state 3 and state 4 respiration rate. Data are means ± SD of 5 experiments performed with duplicate samples.

### Activities of OXPHOS complexes

**Figure 1:**
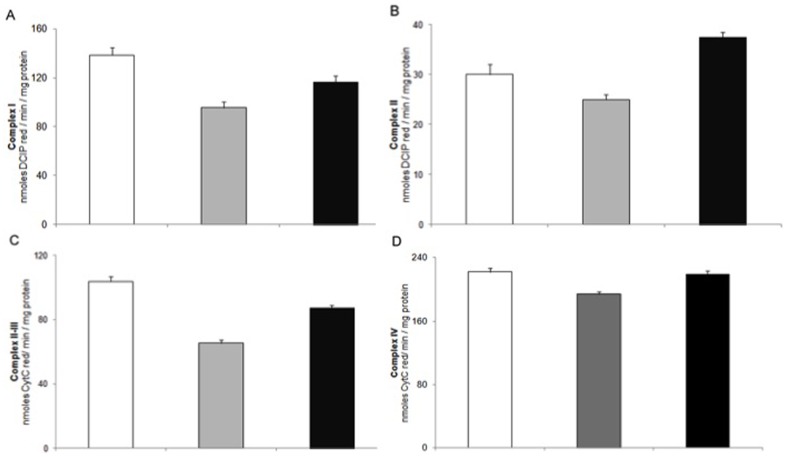
OXPHOS complexes enzyme activities. (A) NADH: coenzyme Q oxidoreductase (Complex I); (B) Succinate: coenzyme Q oxidoreductase (Complex II); (C) Succinate: cytochrome c oxidoreductase (Complex II–III); (D) Cytochrome c oxidase (Complex IV). Euthyroid rats (□), hypothyroid rats ( ) and hypothyroid +T_2_-treated rats (▪). Complex activities were determined as described in Materials and Methods section. Complex I and Complex II activities are expressed as nmoles DCIP reduced/min/mg protein. Complex II–III and Complex IV activity: nmoles cytochrome c reduced/min/mg protein. Data are means ± SD of five separate experiments with duplicate samples.

**Figure 2:**
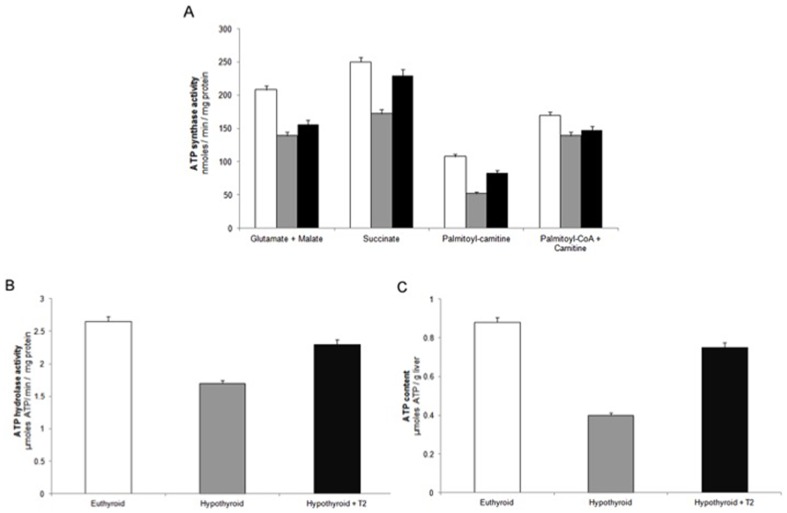
F_o_F_1_ -ATP synthase and hydrolase activities, and ATP content in liver of hypothyroid and T_2_-treated hypothyroid rats. A) ATP synthesis was measured in freshly isolated mitochondria. B) Oligomycin-sensitive ATP hydrolysis was measured in sub-mitochondrial particles prepared in the presence of EDTA (ESMP) and with 1 mM ATP using an ATP-regenerating system. C) ATP content assayed luminometrically as in Materials and Methods. Data are means ± SD (n = 5). Euthyroid rats (□), hypothyroid rats ( ) and hypothyroid +T_2_-treated rats (▪).

Studies on enzymatic activities of mitochondrial respiratory chain complexes were then performed in order to investigate which respiratory complexes are affected by the thyroid state of the animals. The respiratory activities of the four complexes were reduced, as compared to euthyroid, in mitochondria from hypothyroid rats (Fig. 1). In hypo+T_2_ treated rats an increase in the activities of all the complexes was observed; the highest enhancement was detected in complex II activity, which reached values higher than euthyroid mitochondria. Complex IV activity was restored to the euthyroid value by T_2_ treatment. The activity of Complex V was followed in both the directions, i.e. both in the direction of F_o_F_1_-ATP synthase and hydrolase (ATPase) activity. In hypothyroid mitochondria a reduction of ATP synthase activity, particularly evident with succinate or L-palmitoylcarnitine as respiratory substrate, was detected. T_2_ administration to hypothyroid rats reversed in part this reduction (Fig. 2A). To test whether the increased ATP synthase activity observed upon T_2_ treatment could be ascribed to a direct effect of T_2_ on Complex V, the oligomycin-sensitive ATPase activity was assayed in inside-out submitochondrial particles. Consistent with previous data [15], [42] ATPase was greatly reduced in hypothyroid mitochondria and this reduction was in part reversed by T_2_ (Fig. 2B). The ATP level, expressed as µmoles ATP/g liver, was significantly enhanced by T_2_ administration (Fig. 2C).

### T_2_-induced enhancement of fatty acid oxidation rate

**Figure 3:**
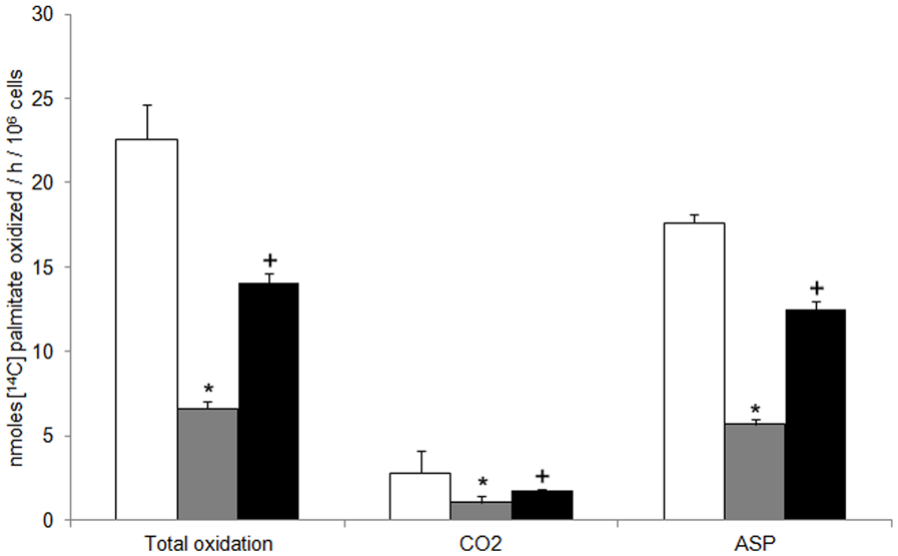
Effect of T_2_ on palmitate oxidation by isolated rat hepatocytes. Hepatocytes were obtained from euthyroid (□), hypothyroid ( ) and hypothyroid +T_2_-treated rats (▪). Hepatocytes were incubated with [1-^14^C]palmitate. Total fatty acid oxidation was obtained as the sum of labeled CO_2_ and total acid soluble products (ASP). Results, expressed as [1-^14^C]palmitate into products/h/10^6^ cells, are means ± SD of 5 experiments with duplicate samples. Values sharing a different symbol differ significantly (P<0.05 vs euthyroid).

Fatty acid oxidation rate, measured starting from [1-^14^C]palmitate, was then followed in intact hepatocytes. Once a fatty acid enters into the mitochondrial matrix its metabolic fate is to be degraded by β-oxidation to acetyl-CoA, which enters the citric acid cycle to be degraded to CO_2_, if the supply of oxaloacetate is sufficient. Alternatively, acetyl-CoA can give rise to ketone bodies. We first examined in our system the rate of β-oxidation flux of labeled palmitate, calculated as radioactivity associated with ASP (mainly ketone bodies), as ^14^CO_2_ release or as total fatty acid oxidation (i.e. the sum of ASP radioactivity and of ^14^CO_2_ release). As shown in Fig. 3 both ^14^CO_2_ and labeled ASP formation were reduced by about 60% in hypothyroid hepatocytes. In hepatocytes from T_2_-treated rats, labeled CO_2_ and ASP formation were enhanced by about 70% and 120%, respectively, as compared to hypothyroid cells. Total fatty acid oxidation rate, strongly reduced (71%) in hypothyroid hepatocytes, noticeably raised (112% vs hypothyroid) in T_2_-treated rats.

### T_2_ rapidly enhances mitochondrial fatty acid uptake

**Figure 4:**
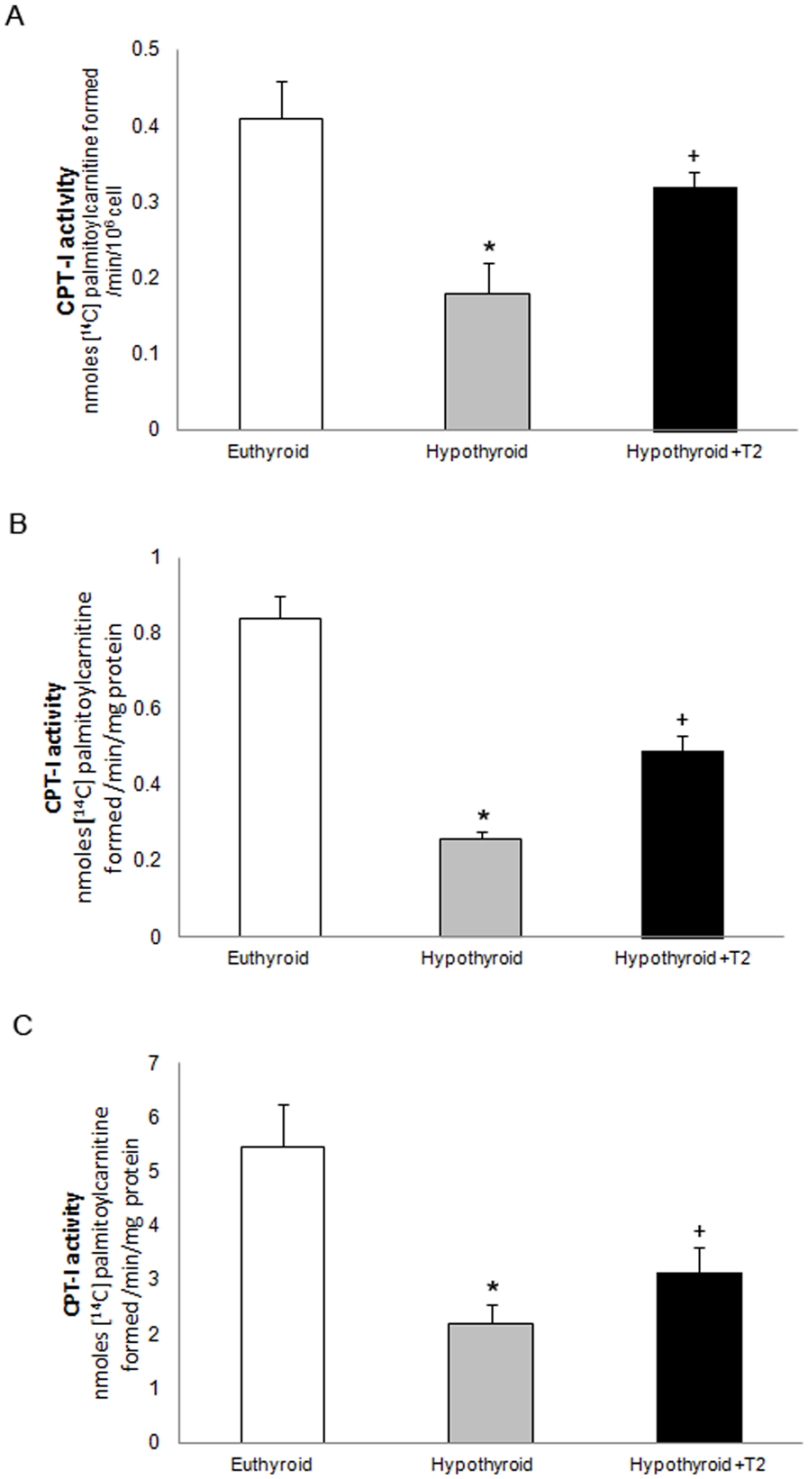
Carnitine palmitoyltransferase I (CPT-I) activity from euthyroid, hypothyroid and T_2_-treated hypothyroid rats (Hypothyroid+T_2_). The CPT-I activity was assayed in permeabilized hepatocytes A), in whole liver homogenate, B) and in isolated liver mitochondria C) by measuring the incorporation of 0.5 mM L-[Methyl-^14^C]carnitine (2 Ci/mol) into [^14^C]palmitoylcarnitine using 50 µM palmitoyl-CoA as substrate. Results are means ± SD of 5 experiments with duplicate samples. Values sharing a different symbol differ significantly (P<0.01 vs Euthyroid).

It is conceivable that the stimulation of fatty acid oxidation observed in hypo+T_2_ treated rats might be attributed to the activity of CPT-I. It has been shown that this enzymatic activity is affected by extramitochondrial cell components that are lost on isolation of mitochondria [37]–[39]. To circumvent this problem, besides the determination of CPT-I activity in whole liver homogenate and in isolated mitochondria, we performed the radioactive assay for determining its activity using [^14^C]carnitine as substrate in digitonin-permeabilized hepatocytes. The reduced (56%) CPT-I activity observed in hypothyroid hepatocytes greatly raised (78%) following T_2_ administration (Fig. 4A), confirming data obtained on tissue homogenate (Fig. 4B) and isolated organelles (Fig. 4C). These findings are consistent with the changes in the rate of [1-^14^C]palmitate oxidation reported in Fig. 3. Collectively, these results indicate that T_2_ increases the ability of mitochondria to import and oxidize fatty acids.

### T_2_ does not influence mitochondria number

**Figure 5:**
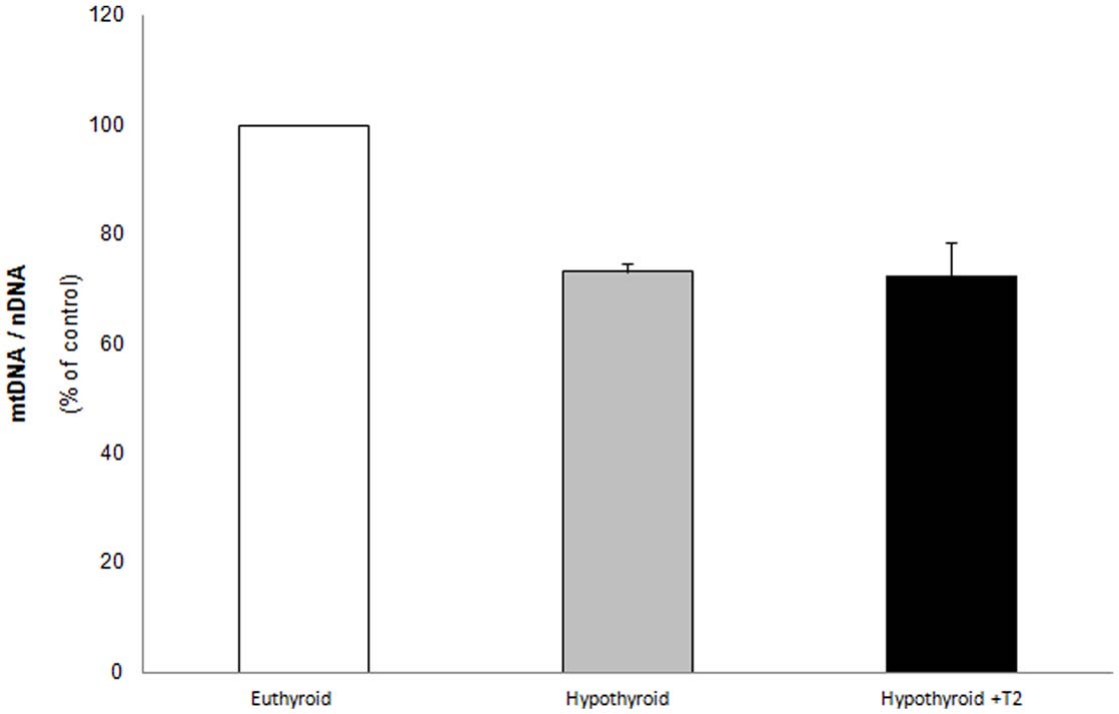
Mitochondria copy number. Total DNA was isolated from liver of control, hypothyroid and hypothyroid+T_2_ treated rats. Quantitative Real time PCR was used to determine nuclear DNA and mtDNA contents by using specific primers, targeting the nuclear gene *Gadph* and the mt D-loop region. The mtDNA level was expressed as mtDNA copy number to the nDNA copy number ratio (mtDNA/nDNA). Data are means ± SD of four experiments (each sample was analyzed in triplet).

To investigate the mechanism of the effect of T_2_ on mitochondrial oxidative capacity, mitochondria number was determined by evaluating mtDNA/nDNA ratio measured by quantitative real time PCR. Data in Fig. 5 show that mitochondria number, significantly decreased in liver of hypothyroid rats, was not affected by T_2_ administration. To our knowledge, these findings represent the first evidence of the influence of animal thyroid state on the liver mitochondria number.

## Discussion

Mitochondria, the major site of the oxidative process in the cell, are considered to be important subcellular targets for thyroid hormone actions [1], [43]. Thyroid hormone effects on respiratory parameters and metabolic pathways are often divided in: i) short-term effects occurring within minutes or a few hours; and ii) long-term effects, occurring over several hours or days [1], [12], [41]. While T_3_ is generally associated with long-term nuclear genomic effects, T_2_ actions are more rapid and occur by direct interaction of the hormone with mitochondria [1], [12], [15], [41].

In the present study we focused on the rapid effect of T_2_ on rat liver, due to the central role of this organ in whole body metabolism. Our results highlight the ability of T_2_, when injected into hypothyroid rats, to induce a rapid (within 1 h) stimulation of mitochondrial OXPHOS activities and of fatty acid oxidation rate in liver cells. Mammalian cells generate most of their ATP by the OXPHOS system, a process located in the inner mitochondrial membrane. Two functional entities carry out this process: first, the respiratory chain, characterised by four Complexes (I, II, III and IV) that transfer electrons to molecular oxygen via electron carriers such as quinone and cytochrome c, and second, the system that phosphorylates ADP to produce ATP, which comprises the F_o_F_1_-ATP synthase. Here, we show that T_2_ administration to hypothyroid rats induces an increase in oxygen consumption rate that was higher when succinate (+rotenone) rather than glutamate/malate was used as respiratory substrate (Table 1). This finding is corroborated by the data reported in Fig. 1, where it is shown that T_2_ enhances in particular Complex II activity, reaching succinate-supported respiration values even higher than those found in euthyroid mitochondria. Our results support those of Lombardi et al. [27] who demonstrated that T_2_ administration to hypothyroid rats rapidly increases in skeletal muscle mitochondria FADH_2_-linked (succinate) respiratory pathways. These authors reported also failure of the T_2_ to significantly affect NADH-linked (pyruvate+malate) mitochondrial respiratory pathways.

Under physiological conditions, maximal respiratory capacity is obtained with glutamate/malate/succinate as substrates, reconstituting the operation of the tricarboxylate cycle in intact cell by the simultaneous generation of NADH and succinate as substrates of Complex I+II [33]. Here, we show for the first time that maximal respiratory activity investigated with such a substrate combination, and strongly reduced in hypothyroid mitochondria, was stimulated by T_2_ administration. A significant enhancement of the F_o_F_1_-ATP synthase was observed particularly when succinate or L-palmitoylcarnitine were provided as respiratory substrate (Fig. 2A). Consistent with previous findings [15], a T_2_-induced raise of oligomycin-sensitive ATPase activity (Fig. 2B), measured in rat-liver inside-out submitochondrial particles, was detected. Hepatic ATP level was also raised by T_2_ injection. Overall, these findings indicate that OXPHOS activities were acutely (within 1 h) stimulated by T_2_ administration to hypothyroid rats. Noticeably, T_2_ treatment also increased mitochondrial oxygen consumption supported by fatty acids. Respiratory activity was higher when palmitoyl-CoA (whose oxidation requires its import into the mitochondria, mediated by the CPT system) was used as respiratory substrate. These findings led us to hypothesize that T_2_ promotes channelling of long chain fatty acids towards mitochondrial β-oxidation. This hypothesis turned out to be true. Indeed, data in Fig. 3 showed a T_2_-induced significant increase in the conversion of [1-^14^C]palmitate to ^14^CO_2_ and ketone bodies, which are considered to be a direct and accurate measure of fatty acid β-oxidation [41], [44]. Our data strengthen previous findings of Cimmino et al. [45] who reported that fatty acid oxidation, measured as ^13^CO_2_ recovered in breath following the injection of [1-^13^C]fatty acid, decreased in hypothyroid as compared with euthyroid animals and restored to control values by T_2_. In liver, CPT-I exerts approximately 80% of control of fatty acid oxidation under physiological conditions [26], [46]. A great body of evidence indicate that hepatic CPT-I activity is modulated on the long term in different physiopathological conditions [11], [36], [46]–[49]. Short-term control events have not received extensive attention due to the fact that short-term modulation of CPT-I activity is difficult to preserve during the time required for cell disruption and subsequent isolation and purification of mitochondria [37], [38]. We avoided this problem with a one-step *in situ* assay for determining CPT-I activity in digitonin-permeabilized hepatocytes. Results of Fig. 4A clearly indicated that CPT-I activity was strongly reduced (∼60%) in hypothyroid hepatocytes. Compared to the latter, a 78% increase in CPT-I activity was observed following T_2_ administration, thus indicating CPT-I activity as a possible target of T_2_ action. A similar trend was observed when CPT-I activity was measured in liver homogenate and isolated mitochondria (Fig. 4B and 4C).

As far as the mechanism of the short-term effect of T_2_ administration to hypothyroid rats is concerned, it has been shown that T_2_ was not able to influence hepatic mRNA level and protein amount of some enzymes of OXPHOS [15]. The results in Fig. 5, showing the ineffectiveness of T_2_ in influencing mitochondria number, are in agreement with this observation.

Different mechanisms, including changes in membrane lipid composition [13], are involved in short-term regulation of metabolic pathways. The activities of several mitochondria proteins are known to be influenced by organelle phospholipid composition, mainly by cardiolipin (CL) level [6], [15], [50], [51]. Due to its almost exclusive location in the inner mitochondrial membrane, CL interacts with a number of inner membrane mitochondrial proteins, including electron transport chain complexes. CL is required for optimal activity of Complex I, III, IV and V [51]. Requirement of CL for CPT-I activity has also been indicated [52]. Moreover, thyroid hormone has been shown to directly modulate the CL content [6], [15], [51]. It is conceivable that changes in membrane environment, because of the formation of localized membrane microdomains of specific lipid composition, due to the action of T_2_, can induce changes in OXPHOS and CPT-I activities. In a recent paper, it has been shown that T_2_ administration to hypothyroid rats, under the same experimental conditions of the present study, rapidly increases mitochondrial cardiolipin content, occurring in parallel with a decreased level of the cardiolipin oxidized form [15].

However, other mechanisms underlying the rapid T_2_ action, such as the involvement of AMP-activated protein kinase in metabolic signalling pathway [27], cannot be excluded. Studies are in progress in our laboratory to clarify this point.

Overall, our results show that T_2_ rapidly increases the ability of rat-liver mitochondria to import and oxidize fatty acids. The enhanced β-oxidation is coordinated with downstream stimulation of pathways such as respiratory chain and OXPHOS complexes.

In the last years, T_2_ use as potential anti-obesity drug has been proposed [21]–[23]. T_2_ has been shown to powerfully reduce adiposity and dyslipidemia, and when administered to rats simultaneously receiving a high-fat diet, can prevent excessive body weight gain and development of liver steatosis, without unfavourable side-effects (i.e. thyrotoxicosis) usually observed when T_3_ or T_4_ is administered [21], [53], [54]. A direct action of T_2_ in reducing the excess fat storage in cultured rat hepatocytes has recently been shown [23]. Our results, showing short-term hepatic enhancement by T_2_ of fatty acid oxidation rate and OXPHOS activities may help towards a better understanding of the biochemical mechanisms underlying T_2_ metabolic effects.
